# Emerging nanophotonic biosensor technologies for virus detection

**DOI:** 10.1515/nanoph-2022-0571

**Published:** 2022-12-01

**Authors:** Shivananju Bannur Nanjunda, Venkatesh N. Seshadri, Chitra Krishnan, Sweta Rath, Sivasubramanian Arunagiri, Qiaoliang Bao, Kristian Helmerson, Han Zhang, Ravi Jain, Asokan Sundarrajan, Balaji Srinivasan

**Affiliations:** Department of Electrical Engineering, Centre of Excellence in Biochemical Sensing and Imaging (CenBioSIm), Indian Institute of Technology Madras, Chennai, India; Department of Instrumentation and Applied Physics, Indian Institute of Science, Bangalore, India; Department of Life Science, Indian Academy, Bangalore, India; School of Electronics Engineering, Vellore Institute of Technology, Chennai, India; Department of Materials Science and Engineering, and ARC Centre of Excellence in Future Low Energy Electronics Technologies (FLEET), Monash University, Clayton, VIC, Australia; School of Physics and Astronomy, ARC Centre of Excellence in Future Low-Energy Electronics Technologies (FLEET), Monash University, Clayton, VIC 3800, Australia; International Collaborative Laboratory of 2D Materials for Optoelectronics Science, College of Physics and Optoelectronic Engineering, Shenzhen University, Shenzhen 518060, China; Optical Science and Engineering Program, Center for High Technology Materials, Departments of ECE, Physics Astronomy, and Nanoscience Microsystems, University of New Mexico, Albuquerque, NM 87106, USA

**Keywords:** diagnostic techniques, healthcare, nano or two-dimensional materials, nanophotonics, optical biosensors, SARS-CoV- 2 (COVID-19), virus detection

## Abstract

Highly infectious viral diseases are a serious threat to mankind as they can spread rapidly among the community, possibly even leading to the loss of many lives. Early diagnosis of a viral disease not only increases the chance of quick recovery, but also helps prevent the spread of infections. There is thus an urgent need for accurate, ultrasensitive, rapid, and affordable diagnostic techniques to test large volumes of the population to track and thereby control the spread of viral diseases, as evidenced during the COVID-19 and other viral pandemics. This review paper critically and comprehensively reviews various emerging nanophotonic biosensor mechanisms and biosensor technologies for virus detection, with a particular focus on detection of the SARS-CoV-2 (COVID-19) virus. The photonic biosensing mechanisms and technologies that we have focused on include: (a) plasmonic field enhancement via localized surface plasmon resonances, (b) surface enhanced Raman scattering, (c) nano-Fourier transform infrared (nano-FTIR) near-field spectroscopy, (d) fiber Bragg gratings, and (e) microresonators (whispering gallery modes), with a particular emphasis on the emerging impact of nanomaterials and two-dimensional materials in these photonic sensing technologies. This review also discusses several quantitative issues related to optical sensing with these biosensing and transduction techniques, notably quantitative factors that affect the limit of detection (LoD), sensitivity, specificity, and response times of the above optical biosensing diagnostic technologies for virus detection. We also review and analyze future prospects of cost-effective, lab-on-a-chip virus sensing solutions that promise ultrahigh sensitivities, rapid detection speeds, and mass manufacturability.

## 1 Introduction

Highly infectious viral diseases – particularly those with debilitating impact on individuals affected – are a serious threat to humans as they can spread rapidly among the community, often leading to serious health consequences including the loss of life. One such example is the recent rapid spread of COVID-19 (coronavirus disease-19), which is caused by severe acute respiratory syndrome coronavirus 2 (SARS-CoV-2) and has severely affected the global community during the past 4 years.

Preventive measures such as personal hygiene, use of personal protective equipment (PPE) and disinfection methods can help reduce the virus spread [1]. However, once such an outbreak has occurred, early detection of the infected people is of paramount importance so that all infected individuals can be isolated and the chain of spread may be broken efficiently. The asymptomatic spread of COVID-19 pandemic as well as its long-term impact due to the frequent emergence of new mutants (such as the delta and omicron variants) necessitates the development of rapid, accurate, and highly reliable point-of-care diagnostic technologies that will help combat the spread of such outbreaks effectively.

There are two broad approaches for viral detection: (1) direct detection of the virus (a) by identifying critical genetic material, namely the fundamental nucleic acids (DNA/RNA) constituting the virus [1] or (b) by identifying the proteins present on its surface, collectively called antigens, and (2) indirect direction through clinical (including serological) methods that rely on the detection of antibodies in the host in response to the virus.

A direct detection approach based on reverse transcription polymerase chain reaction or RT-qPCR is the most sensitive and specific method of viral RNA detection to date. It detects the viral RNA by replicating the RNA polymerase (RdRp) sequence, the genetic marker extracted from samples collected through respiratory secretions [2]. RdRp of all known double-stranded RNA viruses is also responsible for the transcription and replication of the viral genome [3]. Detection of other genetic markers for viruses, especially for SARC-CoV2 virus, can also be performed using various molecular methods and genome sequencing methods [4].

However, since RT-qPCR and genome sequencing methods require expensive laboratory infrastructure, as well as highly trained personnel for processing and analysis of the samples, their use is impractical from the perspective of large-scale identification of infectious individuals during a pandemic. A good example for an indirect detection approach is the one based on antigen-directed diagnostics. In this approach, the virus is detected using specific antibodies such as Immunoglobulin G (IgG) and Immunoglobulin M (IgM). These antibodies capture the S (spike) protein present in coronavirus, and the HA (hemagglutinin glycoprotein) protein in viruses. In contrast, the detection through serological methods involve analysis of the patient’s blood or saliva and identification of antibodies through the capture of viral antigens. Conventional methods such as ELISA used for antigen/antibody detection provide good accuracy, but also require well-trained skilled personnel to perform the testing and results analysis, and are laborious as well as time-consuming [5]. Immuno-chromatography or lateral flow assays based rapid diagnostic tests (RADT) are relatively inexpensive, provide quick turnaround times, and point-of-care (PoC) testing capabilities, but have very limited sensitivities and poor positive predictive values [6].

In general, the utility of the above biomolecular diagnostic approaches is limited in both acute and early stages of infection, since it typically takes about a week after being infected for enough antibodies (Ig-G and Ig-M) to develop to quantities and densities that are high enough to be detected easily and accurately; as such, these tests can potentially yield false negative results at unacceptable rates. Therefore, more reliable, rapid and less equipment-intensive easy-to-use virus detection methods are critical for virus detection and control during pandemics; emerging optical and photonic nanophotonic technologies hold strong promise as biosensors in this regard.

## 2 Optical sensors for virus detection

A typical biosensor consists of (a) a bio-receptor unit for “receiving” or binding a particular analyte (virus) of interest, and (b) a transduction mechanism or methodology for converting information on the number of bound target virus/biomolecules (“analytes”) into a quantifiable value by means of a measurable electrical, optical, or thermal signal [7–9]. Based on the transduction method used and the physical principles involved, biosensors can be classified into several categories, such as electrochemical, thermal, piezoelectric, and optical/photonic. Among the different types of common biosensors, electrochemical and optical biosensors are the most widely used [10, 11]. The operation of electrochemical biosensors is based on the direct generation of voltages and currents that are proportional to the analyte concentration, whereas optical or photonic biosensors may use a series of steps to get an optical or electronic signal that is indicative of the analyte concentration.

Optical biosensors are advantageous over electrochemical biosensors since they usually provide much higher levels of sensitivity, selectivity or specificity, along with a capability of faster detection response, stability, immunity to electromagnetic interferences (external disturbances), amenability to miniaturization and integration capabilities, leading to portable devices [12–25]. As such, optical biosensors are excellent candidates to move diagnostic technologies from centralized laboratories to point-of-care. Furthermore, detection of analytes can be achieved at femtogram levels and virus detection has been demonstrated even at the single molecule level by using optical biosensors [25].

**Figure 1:**
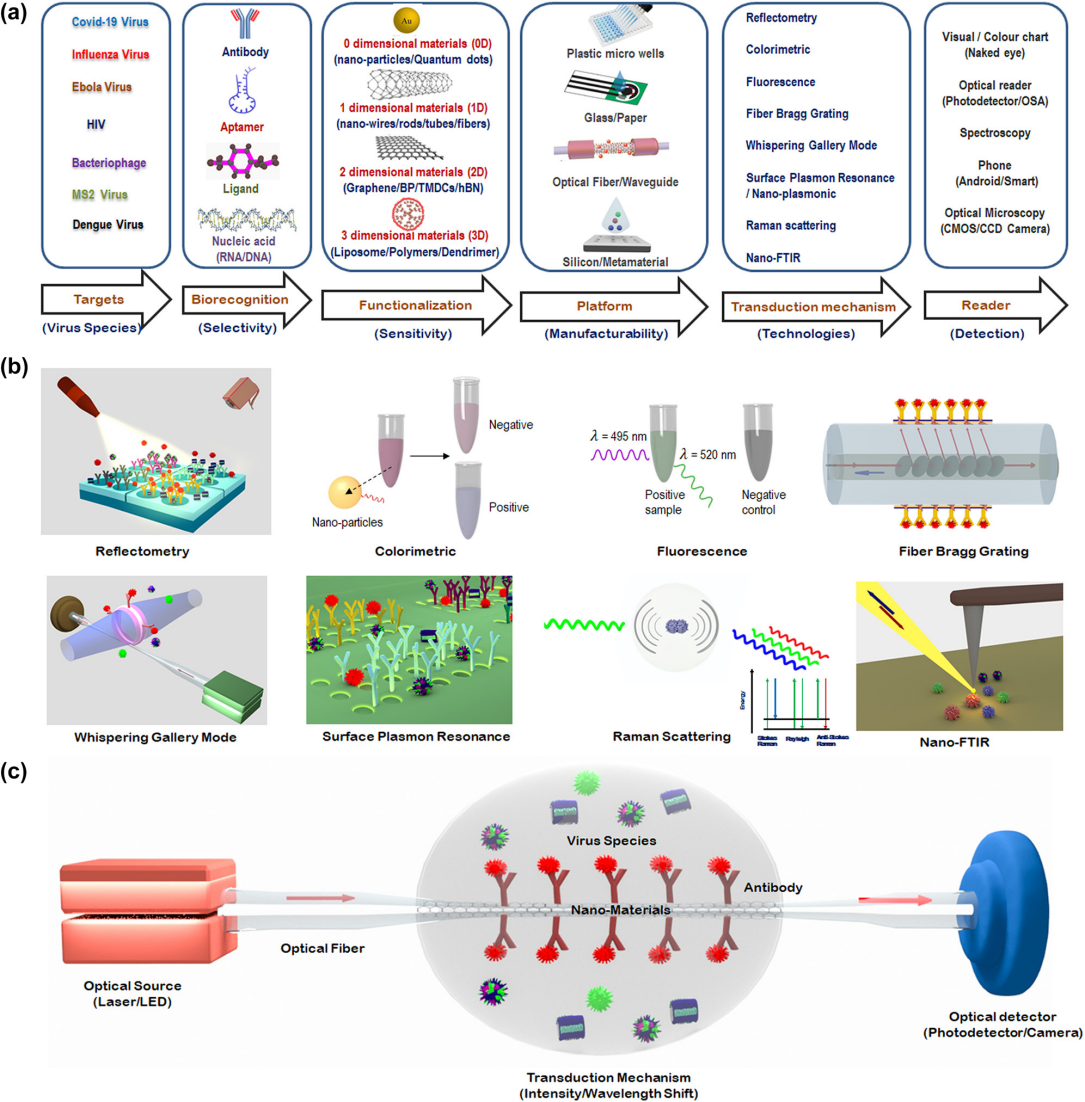
Schematic depiction of optical biosensing methods for virus detection. (a) Various steps involved in choosing an optical biosensor for virus detection. (b) Visual illustration of some of the most common optical transduction mechanisms, with a focus on the mechanisms of greatest relevance to detection of virus variants relevant to COVID-19. (c) Schematic diagram of a typical optical diagnostic for virus detection.

The various steps involved in the development of an optical biosensor for biomolecular (virus) detection are schematically shown in Figure 1(a). The first step is to define the target biomolecular species/analyte to be detected, followed by the determination of the biorecognition mechanism, which is key not only to achieving the desired selectivity but also to finalizing the choice of the specific photonic technology to be employed. As illustrated in Figure 1(a), the next step typically involves functionalizing the biorecognition element on an appropriate platform (such as in microwells, on microresonators, fiber tips or glass/paper/silicon/metamaterial substrates) based on the choice of the biotransduction mechanism to be employed. Figure 1(b) illustrates the various transduction mechanisms or technologies used for virus detection both schematically and visually, and Figure 1(c) shows a “simple” or “baseline” optical measurement setup schematically, which constitutes illumination of the biorecognition element before and after its interaction with the analyte, and a methodology for detecting changes to the light beam as a consequence of the interaction of the biorecognition element with the analyte.

In general, the optical biosensor typically consists of an optical source, a transduction platform, and an optical detector/receiver as shown schematically in Figure 1(c). The transduction platform contains appropriate receptors designed to bind specifically with the target analytes (virus). Out of the different transduction platforms elucidated in Figure 1(b), optical fiber-based platforms are particularly attractive since they are capable of performing well in harsh environments and their amenability to array sensing enables their use for multichannel and multiparameter detection [26].

The three fundamental effects employed in all the transduction mechanisms used for biosensing resulting from an interaction between the light wave (emitted by the optical source in Figure 1(c)) and the target analyte (virus) are: (a) a change in the phase (or the refractive index) experienced by the incident light wave; (b) a change in the amplitude or intensity of the light wave due to the absorption of the energy of the incident light wave by the biomolecule; or (c) emission or generation of new wavelengths of light subsequent to the energy absorption in the biomolecules by linear or nonlinear processes such as fluorescence or Raman scattering. By careful engineering of optical devices on the substrate platforms, optical phase shifts can be converted to wavelength shifts (by means of photonic devices such as microresonators, and fiber Bragg gratings) or to light intensity changes (through interferometers). ***Practical optical biosensors are engineered by designing substrate platforms and devices that detect changes in the optical properties of the light, typically the light intensity, phase or wavelength with the requisite amount of*** ***precision*** ***.***

Based on the transduction mechanism involved, optical biosensors may be broadly classified as amplitude/intensity modulated or wavelength/frequency modulated sensors. One of the key drawbacks of the amplitude/intensity modulated sensors is that they are susceptible to noise, which compromises their ability to quantify the precise concentration of the viral particles. In contrast, *if viral concentrations are determined by measuring changes in the wavelength of light, the measurements are much less likely to be corrupted by noise since the typical noise sources do not change the wavelength of light.* Hence, we have chosen to prioritize discussing the various emerging wavelength modulated optical biosensing techniques for virus detection in the following section.

## 3 Transduction mechanisms for the most promising nanophotonic biosensors

As discussed in the previous section, optical biosensing techniques based on wavelength modulation are often much more attractive for virus detection since they are relatively less prone to noise. In this section, we describe the transduction mechanism for some of the emerging nanophotonic biosensors for virus detection.

### 3.1 Surface plasmon resonance

Plasmonic sensors are used for extremely sensitive detection of biological molecules, including viruses [27–33]. The excitation of noble metal nanostructures with light produces surface plasmons (SPs) due to the collective oscillation of conduction electrons of metal that contribute to optical phenomena such as localized surface plasmon resonance (LSPR), and extraordinary optical transmission (EOT). The most intense plasmonic fields usually appear within narrow gaps between adjacent metallic nanostructures, especially at nanometer and sub-micron scales and hence are well suited for detecting ultrasmall viral species.

The change in the refractive index at the sensor surface [34] produced by the capture of biomolecules depends on the concentration of analyte molecules (*c*) within a thin layer at the surface (*h*). The limit of detection (LoD) depends on the minimum resolvable change of molecular mass of the analyte (*σ*) as given in Eq. (1):

(1) $$\sigma =\sigma_{\text{RI}}\frac{L_{\text{pd}}}{2{\left(\frac{\partial n}{\partial c}\right)}_{\text{vol}}}$$

where *σ*_RI_ is refractive index resolution, *L*_pd_ is surface plasmon penetration depth and ${\left(\frac{\partial n}{\partial c}\right)}_{\text{vol}}$ is the volume refractive index change with respect to molecular concentration of the analyte [34].

**Figure 2:**
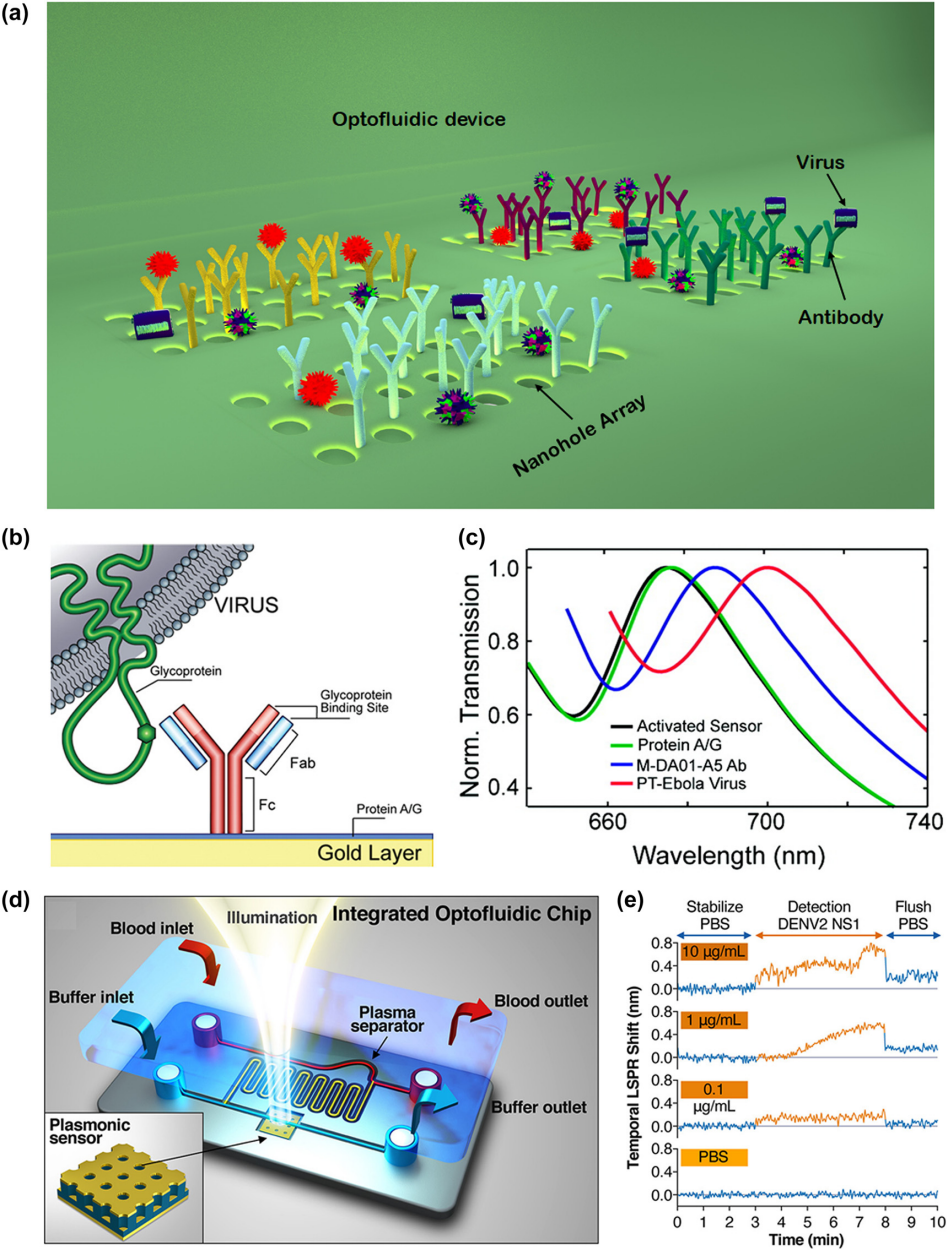
Illustration of the optofluidic nano-plasmonic technique for virus detection. (a) Three-dimensional illustration of the detection scheme using optofluidic nano-plasmonic biosensors based on resonance transmissions due to extraordinary light transmission effect. (b) Illustration of the immunosensor surface functionalization. Antiviral immunoglobulins are attached from their *F*_c_ region to the surface through a protein A/G layer. Adapted with permission from ref. [35]. Copyright [2010] American Chemical Society. (c) Binding results in large effective refractive index increase leading to a strong red shift of the plasmonic resonance. Adapted with permission from ref. [35]. Copyright [2010] American Chemical Society. (d) Integrated optofluidic device for Dengue virus detection based on nanoimprinted plasmonic array. Adapted with permission from ref. [42]. Copyright [2021] American Chemical Society. (e) Temporal evolution of the LSPR shift for three DENV2-NS1 protein concentrations (0.1, 1, and 10 μg/mL) in PBS and a control sample (no proteins in PBS). Adapted with permission from ref. [42]. Copyright [2021] American Chemical Society.

One good example that illustrates the potential of SPR-based sensors is the optofluidic nanoplasmonic approach [35]. The sensing mechanism is based on the EOT of light through subwavelength nanohole arrays due to the coupling of normally incident light to the SPs formed around the nanoholes at the metal-dielectric interface (Figure 2(a)) [35–41]. The EOT resonance occurs at a specific wavelength that depends on the dielectric constant of the medium around the sensor. As pathogens bind to the metal surface or to the ligands immobilized on the metal surface, the effective refractive index of the medium increases, causing a redshift of the resonance wavelength indicating the presence of virus.

The optofluidic nanoplasmonic biosensor [35, 36] is capable of detecting virus at concentrations ranging from 1 to 10 μM. The sensor utilises group-specific antibodies for detection of various intact virus strains. The sensor detects small enveloped RNA viruses (vesicular stomatitis virus (VSV) and pseudotyped (PT) Ebola virus) as well as large enveloped DNA viruses (vaccinia virus). To immobilize virus-specific antibodies for specific detection of viruses, the sensor surface is first coated with protein Albumin/Globulin (A/G). It has strong affinity towards fragment crystallizable (*F*_c_) region of antibodies and is responsible for the proper orientation of antibodies (Figure 2(b)). Specific antiviral immunoglobulins targeting the viral glycoproteins are then spotted on the array. In particular, VSV virus with 10^8^ PFU/mL concentration has been demonstrated to produce a strong redshift of ∼100 nm whereas unfunctionalized sensor produces a negligible redshift of ∼1 nm due to nonspecific binding (Figure 2(c)). This strong spectral shift changes the colour of the transmitted light, which can be observed visually, supporting point-of-care diagnostics. For PT-Ebola and Vaccinia virus detection, 10^8^ PFU/mL concentrations of each are added on 12 sensors (9 functionalized and 3 unfunctionalized reference sensors). Spectral redshifts of ≥14 nm (ranging from 14 to 21 nm) are observed for all the 9 functionalized sensors which shows repeatability of measurement whereas negligible shifts for reference sensors.

The limit of detection (LoD) of the sensor is 0.05 nm, which shows the potential of the sensor to detect the virus with at a concentration as low as 0.7 ng/mL [43]. However, background shifting due to non-specific binding can influence the resultant output shift at low concentration of analyte, which can be reduced by capping the unreacted protein A/G [35].

Vázquez-Guardado et al. [42] demonstrated an integrated hybrid microfluidic-plasmonic device (Figure 2(d)) for direct detection of dengue virus in blood sample through capture of its non-structural protein NS1. Figure 2(e) shows LSPR shift at three different NS1 protein concentrations (0.1, 1, and 10 μg/mL) demonstrating the limit of detection of the device at a concentration of 0.1 μg/mL.

Note that the above nano-plasmonic biosensing platform can be extended for SARS-2 detection with appropriate surface functionalization of the optical/photonic sensor, which improves sensitivity, specificity, and detection speed.

### 3.2 Surface enhanced Raman scattering

**Figure 3:**
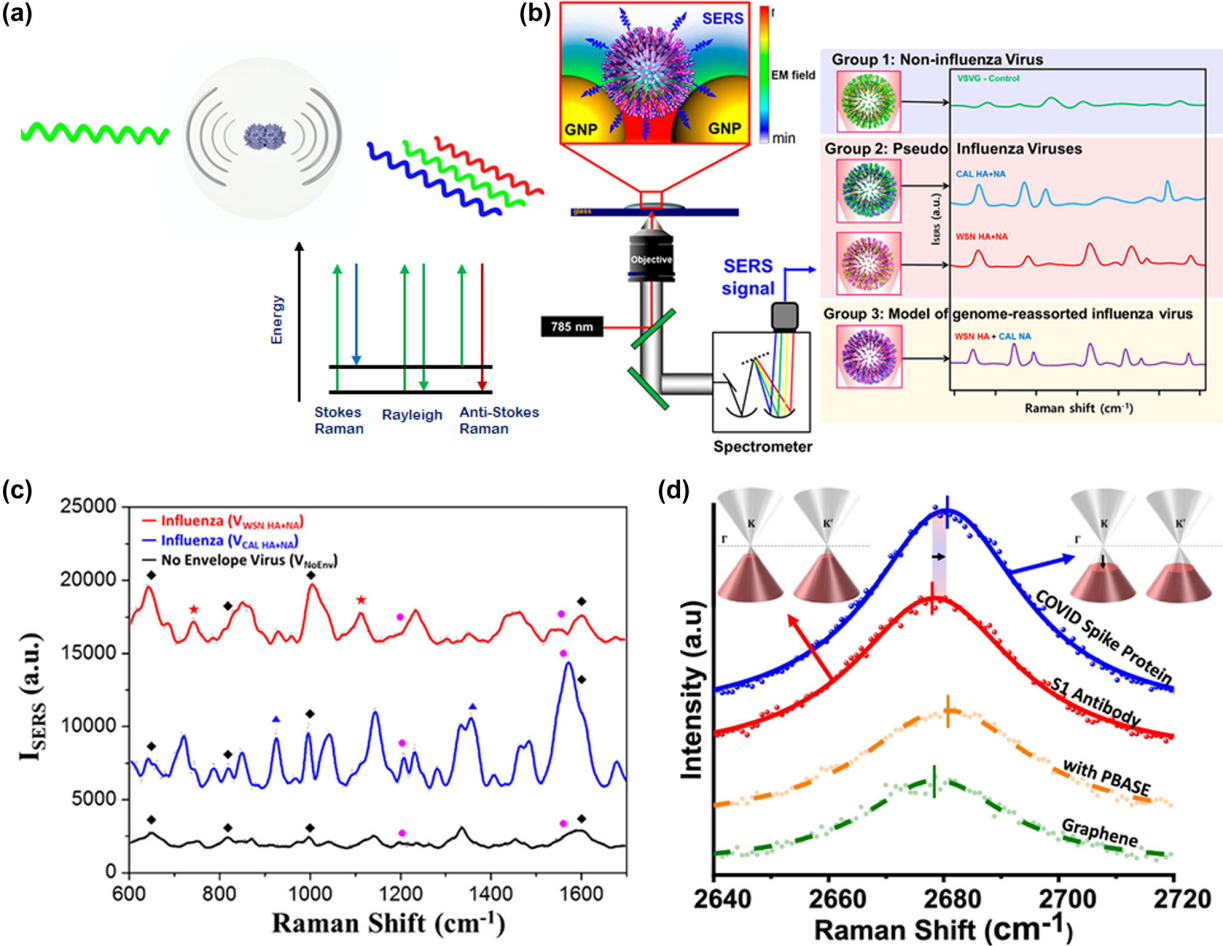
Illustration of the Surface Enhanced Raman Scattering (SERS) technique for virus detection. (a) Concept of detecting virus using Raman scattering due to the molecular vibrations. (b) Schematic diagram representing the identification of viruses by Surface Enhanced Raman Scattering (SERS) method. Adapted with permission from ref. [49] Copyright [2015] American Chemical Society. (c) Raman spectra of V_CAL HA + NA_ (a newly emerging influenza virus strain) and V_WSN HA + NA_ (a lab adapted influenza virus strain). Adapted with permission from ref. [49]. Copyright [2015] American Chemical Society. (d) COVID-19 spike protein detection via graphene phononics in artificial saliva media. Adapted with permission from ref. [52]. Copyright [2021] American Chemical Society.

Surface enhanced Raman scattering (SERS) is an extension of the SPR phenomenon discussed in the previous section. Upon laser excitation on gold nanoparticles (GNPs), the SERS effect causes localized field enhancement around gold nanostructures [43]. When the nano-scale surface proteins and lipids present on envelope of the virus come in contact with these metal nanoparticles, enhanced unique Raman scattering spectra are obtained due to the molecular vibrations (Figure 3(a)) characteristic of the virus [43–51].

Froehlicher and Berciaud [53] studied the electron-phonon coupling in pristine graphene. Raman spectra indicated a strong coupling between zone-centre phonons and low-energy electronic excitations of graphene leading to a shift in frequency (∆ω) which is proportional to the change in Fermi energy (*E*_F_) of graphene as given in Eq. (2):

(2) $$\Delta \omega \approx \frac{D}{2\pi h}\left|E_{\text{F}}\right|$$

where *D* is the dimensionless coefficient corresponding to phonon–electron coupling strength, and *h* is the reduced Planck’s constant. Spectral measurements with different concentrations of the analyte may be recorded and the LoD corresponds to the minimum concentration that provides a noticeable shift in frequency.

Lim et al. [49] proposed SERS method for real-time, rapid, label-free, and precise detection of newly emerging viruses and their mutants (Figure 3(b)). They hypothesize that every virus (enveloped) has a set of lipids and proteins, which are unique and can produce unique Raman scattering spectra. The SERS substrate is fabricated using salt induced GNPs aggregation method. Four different viruses, one non-influenza virus, two influenza viruses, and one genetically shuffled influenza virus are taken for SERS measurement. As observed from Figure 3(c), Raman spectra of influenza virus, V_WSN HA + NA_ produces two peaks common to all of the viruses at 1231 cm^−1^ and 1587 cm^−1^ and two distinct peaks at 740 cm^−1^ and 1107 cm^−1^ from that of V_CAL HA + NA_, which shows unique spectral characteristics of two different influenza viruses. This proves that SERS can be used to distinguish not only non-influenza viruses from influenza viruses but also one influenza virus from another. Also, newly emergent mutant influenza viruses can be detected in a label-free manner, which is not possible by conventional labelled techniques such as ELISA and RT-PCR.

Another example use of Raman spectroscopy for specific viral detection is presented by Nguyen et al. [52], where selective detection of COVID-19 virus is achieved by using antibody functionalized two-dimensional (2D) graphene material as Raman transducer platform. Since graphene’s phononics are strongly sensitive to the change in its doping level induced by the attachment of analyte molecule, binding of negatively charged COVID-19 spike receptor binding domain (RBD) protein on CoV-2 spike RBD antibody functionalized graphene induces p-doping of the p-type graphene which leads to a blue shift in 2D phonon vibration mode peak (Figure 3(d)). The achieved LoD with this 2D material-based phononic sensor is 3.75 fg/mL and 1 fg/mL of s-protein in artificial saliva and PBS solution, respectively.

### 3.3 Nano-Fourier transform infrared spectroscopy

Nano-Fourier Transform Infrared (nano-FTIR) spectroscopy may also be considered as an extension of the SPR phenomenon. Nano-FTIR is a powerful combination of (a) a scattering type scanning near-field optical microscope (s-SNOM) nano-imaging system which consists of a nano metallic (gold or silver) probe tip with (b) broadband wavelength (tunable quantum cascade laser (QCL) or a broadband mid-infrared laser) illumination and FTIR-based detection methods [54].

**Figure 4:**
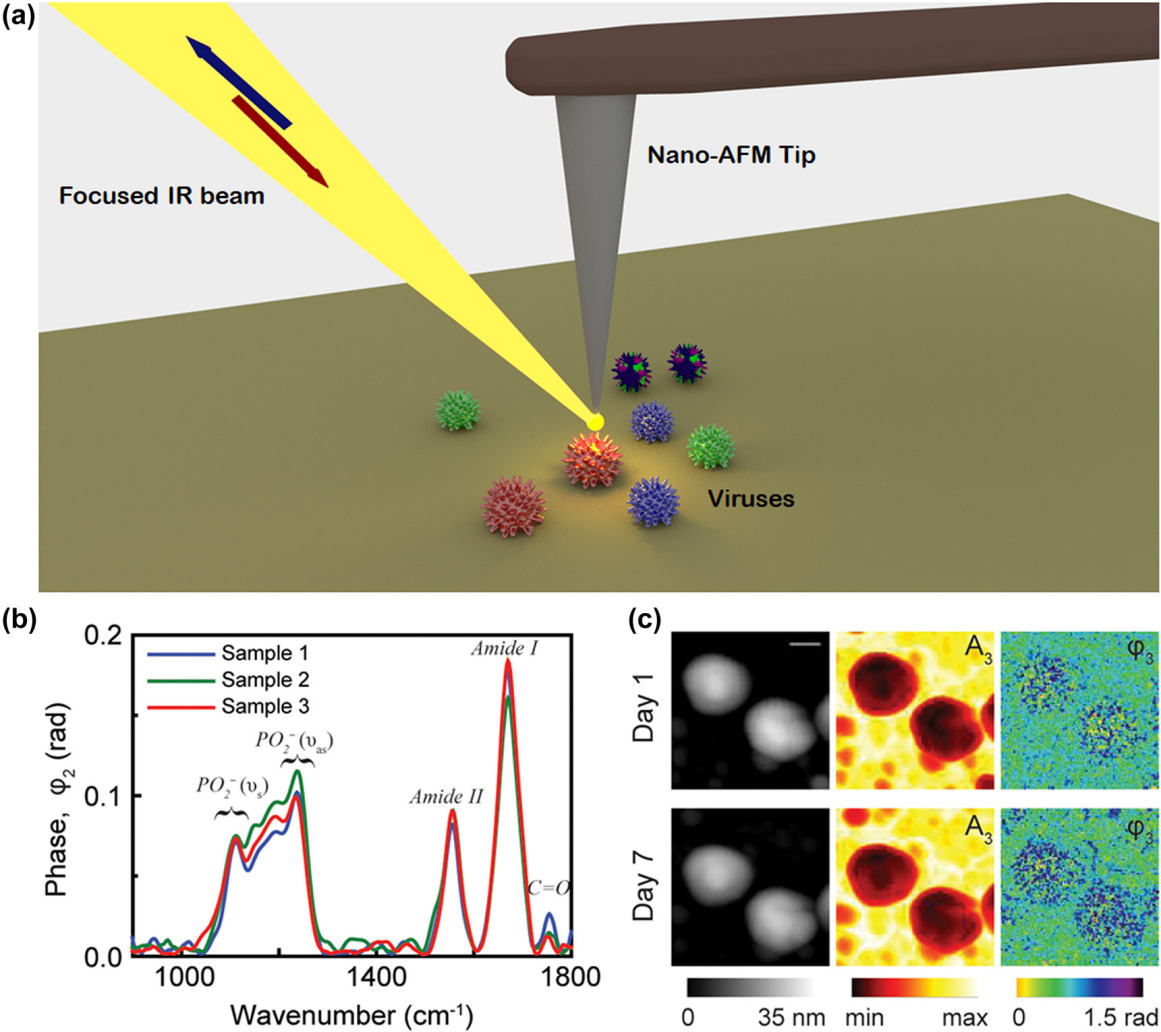
Illustration of the nano-Fourier transform infrared (nano-FTIR) technique for virus detection. (a) Schematic diagram of the s-SNOM experimental setup. (b) Nano-FTIR spectra of an influenza virus particles from three different samples at neutral pH demonstrating the reproducibility of nanoscale analysis. Adapted with permission from ref. [55]. Copyright [2018] PLOS. (c) Topography, near-field amplitude (A3) and phase (*φ*3) images representing reflectivity and absorption of two influenza virus particles on day 1 and day 7. Scale bar 100 nm. Adapted with permission from ref. [55]. Copyright [2018] PLOS.

Upon illumination by focused IR laser beam on the metalized sharp nano-atomic force microscopy (nano-AFM) tip creates a strong nano-focus at its apex as shown in Figure 4(a). The nano-focus acts as an ultra-small broadband light source that probes spectral properties of a sample through near-field optical interaction, which modifies tip-scattered light. During scanning, when the sample containing virus particles comes into this near field, the optical near-field interaction between the tip and the sample modifies the backscattered light creating a signal, which contains the local information of the sample (nanoscale chemical identification and structural imaging of the virus) [54–63]. Nano-FTIR spectroscopy and imaging are performed by detecting the backscattered light interferometrically to precisely obtain IR amplitude and phase spectra, as elaborated below. As the tip and the sample are located in one of the interferometer arms (in contrast to standard FTIR), both local amplitude and phase spectra, *s*_*n*_(*ω*) and *φ*_*n*_(*ω*) can be inferred and normalized to “baseline” references, *s*_ref,*n*_(*ω*) and *φ*_ref,*n*_(*ω*), obtained on a clean area on the sample support. These normalized amplitude and phase spectra are used to calculate the nano-FTIR absorption spectrum *a*_*n*_(*ω*) = *s*_*n*_/*s*_ref,*n*_ sin(*φ*_*n*_ − *φ*_ref,*n*_), which reveals the local infrared absorption spectral response of the virus sample [63]. The virus’ heights are determined from the topographic image and the vibrational IR absorption of the sample (to help identify the biomolecule or the viral components) is obtained from near-field amplitude and phase spectra. The nano-AFM tip maps a single virus at a spatial resolution of 10 nm [63].

With the aim of studying the detailed membrane fusion process involved during virus entry into the host cell, Gamage et al. [55] used single-particle investigation technique to map a single virus. Nano-infrared spectroscopic and imaging measurements (Figure 4(b) and (c)) performed on individual enveloped virus influenza X31 detects the structural and chemical modifications of the viral membrane during environmental pH variations prior to membrane fusion as well as monitors the effect of anti-viral compound (Compound 136) in stopping those membrane modifications due to pH variations.

Figure 4(b) shows the amide I and II bands of absorption centered around 1551 cm^−1^ and 1658 cm^−1^ whereas the spectral band in the range 1290–1050 cm^−1^ depicts the RNA and the lipid bilayer signature. The amide I and II absorption bands remain nearly similar before and after acid treatment while the hyperspectral image taken on specific points of the virus surface in the spectral band 1290–1050 cm^−1^ exhibits significant spectral changes upon acid treatment. The disintegration of RNA and the lipid bilayer at low pH is evident from the spectral changes, which clearly demonstrates disruption of viral membrane.

Figure 4(c) shows a topography image of viruses with height in the range ∼20–30 nm and diameter ∼70–100 nm. The structural modifications of the virus induced at low pH environments are monitored from near-field phase (*φ*3), amplitude (A3) and topography images taken at two laser frequencies (1225 cm^−1^ and 1665 cm^−1^) at pH = 5 and 7 which evidenced gradual disruption of the virus starting from the membrane edges at lower pH. Thus, by using nano-FTIR technique, real time monitoring of the structural and chemical modifications of single influenza [55] or any other virus (COVID-19) can be identified at sub-nanometer (<10 nm) scale resolution overcoming the diffraction limit of conventional optical spectroscopic techniques [63].

### 3.4 Fiber Bragg gratings

Fiber Bragg grating (FBG) sensors consist of a periodic modulation in the refractive index of the core of a single mode optical fiber. When light is guided along the core of the FBG, it gets reflected by successive grating planes; the contributions of reflected light from different grating planes add constructively for a particular wavelength (*λ*_B_), if the following Bragg condition is satisfied: *λ*_B_ = 2*n*_eff_ Λ, where *n*_eff_ is the effective refractive index of the core and Λ is the grating periodicity. In strain or temperature sensing, the grating periodicity (Λ) and/or the effective refractive (*n*_eff_) index changes with strain/temperature, causing a shift in the Bragg wavelength (*λ*_B_) [64].

Recently, FBG sensors have been explored for various applications [64–71] including selective binding of the target analytes or biomolecules detection [66]. The limit of detection (LoD) of optical biomolecules detection techniques is often given in terms of minimum detectable refractive index units (RIU) [65]. One way of enhancing the sensitivity of such FBG biosensors is by ***etching*** of the cladding region to access the evanescent field around the core region to make the FBG more responsive to the minute refractive index changes induced by attachment of the target analytes to the bioreceptors. The change in the cladding layer refractive index can be calculated using Eq. (3),

(3) $$n_{\text{eff}}=n_{\text{clad}}\left[\left(\frac{n_{\text{eff}}^{2}-n_{\text{clad}}^{2}}{n_{\text{core}}^{2}-n_{\text{eff}}^{2}}\right)\left(\frac{n_{\text{core}}-n_{\text{clad}}}{n_{\text{clad}}}\right)+1\right]$$

where *n*_eff_ is the effective refractive index of the core, *n*_clad_ is the refractive index of the cladding, and *n*_core_ is the refractive index of the core [66].

**Figure 5:**
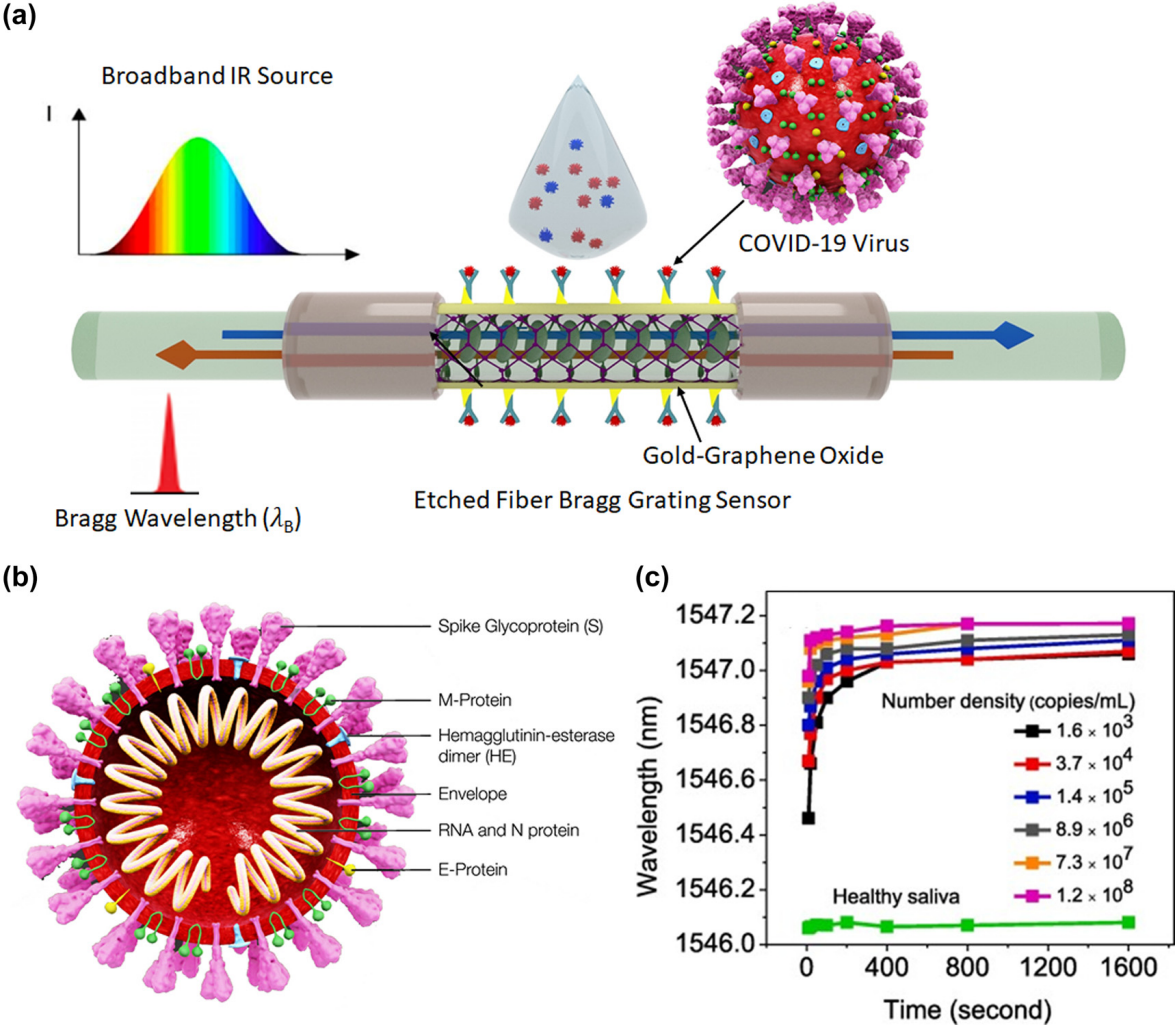
Illustration of the Fiber Bragg Grating (FBG) technique for virus detection. (a) Schematic diagram of the sensing mechanism of etched Fiber Bragg Gratings (EFBG), in which binding of the spike glycoprotein of COVID-19 virus with activated gold/graphene oxide (GO) nanolayer causes a shift in the Bragg wavelength. (b) Schematic diagram of COVID-19 virus with four structural proteins and single-stranded RNA [73]. (c) The wavelength of the detected light after passing through the fiber probe versus exposure time for various virus concentrations. Healthy saliva’s data can be considered as reference, and the amount of deviation from that indicates infection level of the patient. Adapted with permission from ref. [72]. Copyright [2020] Elsevier.

Such etched fiber Bragg grating (EFBG) sensors – based on the evanescent wave interaction with the surrounding medium – have been used successfully for real time detection of various biochemical molecules [66, 72]. This interaction may also be enhanced by the use of “tilted” EFBGs (see Figure 5(a)). The sensitivity can be further enhanced through the coating of nano or 2D materials on EFBG sensor surface which excites Surface Plasmon Resonance (SPR) waves at the cladding interface for virus detection, as illustrated in Figure 5(a). The ultrahigh sensitivity of SPR resonance frequencies to changes in the refractive index induced by the attachment of the analyte to the bioreceptor alters the reflected or the transmitted FBG wavelength spectrum.

The Bragg wavelength sensitivity (*S*_w_) of the SPR-based FBG biosensor is given in Eq. (4):

(4) $$S_{\text{w}}=\frac{\Delta \lambda_{\text{B}}}{\Delta \eta }$$

where Δ*λ*_B_ is the resonance Bragg wavelength shift, and Δ*η* is the density of viruses (copies/mL) [72].

Samavati et al. [72] have described the use of 2D material graphene oxide (GO) decorated gold film (Au)/EFBG sensor for rapid and cost-effective detection of COVID-19 virus from a patient’s saliva. As shown in Figure 5(a), when the light wave reflected from the Bragg grating interacts evanescently with the gold (Au) film, it excites a surface plasmon wave at the Au-graphene oxide (GO) interface. Since this SPR phenomenon is highly sensitive to the refractive index of the surrounding medium, even small changes in the refractive index of this medium can cause a measurable change in the Bragg resonance condition as deduced by ultraprecise measurements of the reflected Bragg wavelength (*λ*_B_). Thus, in this biosensing platform and related transduction mechanism, the sensitivity of the measurement and LoD are limited solely by the wavelength measurement resolution of the spectrometer (or transduction thereof, say by narrow linewidth laser sources and optical edge filters) used for this measurement.

The COVID-19 virus comprises four structural proteins (spike, envelope, membrane, and nucleocapsid) and single-stranded RNA (Figure 5(b)). For detection of COVID-19 virus, the S-glycoprotein consisting of both carboxylic acid group (–COOH) and amine group (–NH_2_) is considered as target protein. When the sensing region comes in contact with the patient’s saliva, the carboxylic acid groups present on the GO’s surface forms a strong covalent-bond with free amine (–NH_2_) groups of S glycoprotein using (1-ethyl-3-(3-dimethylaminopropyl)carbodiimide hydrochloride) EDC/(N-hydroxysuccinimide) NHS as linker molecule. This leads to the formation of a layer of trapped viruses on the surface of GO which changes the local refractive index, leading to a corresponding shift in the Bragg wavelength of the FBG (Figure 5(c)). This shows that the proposed sensor can be used for highly sensitive COVID-19 virus detection with 1.6 × 10^3^ copies/mL in the patient’s saliva within 10 s after exposure of the patient, and this sensitivity can be used for diagnostics in any stage of the disease [72].

### 3.5 Microresonators and whispering gallery modes (WGMs)

**Figure 6:**
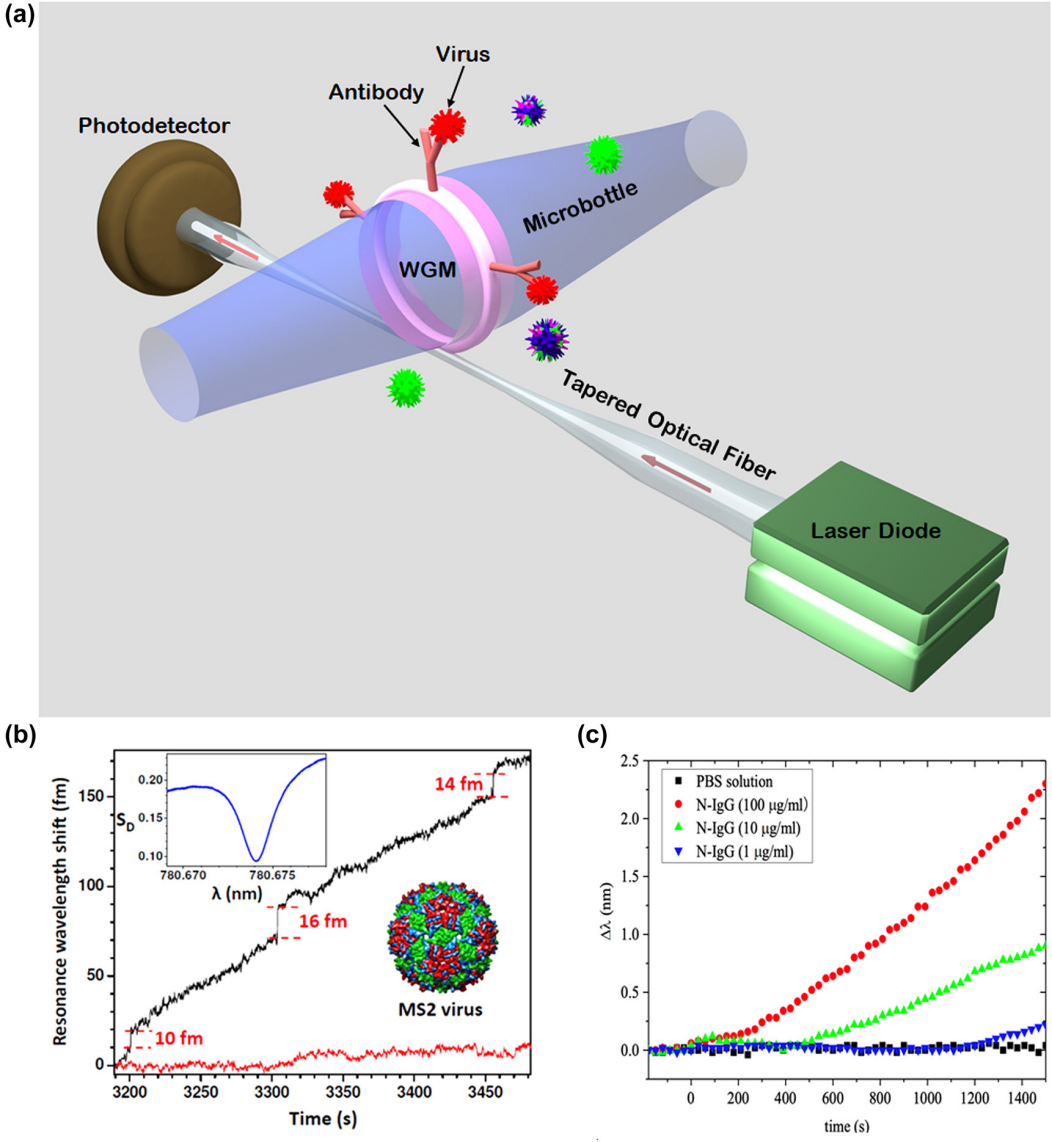
Illustration of the Whispering Gallery Mode (WGM) technique for virus detection. (a) Concept of detecting single virus in a microresonator-based whispering gallery mode (WGM) biosensor. (b) Resonance shift of a WGM resonator of radius 45 μm with gold nanoshell at its equator due to MS2 virus adsorption (upper trace) and background in the absence of nanoshell or MS2 virus (lower trace). Adapted with permission from ref. [77]. Copyright [2012] AIP Publishing. (c) Resonance shift of a WGM microsphere for detection of COVID-19 virus infection through capture of IgG antibodies with different concentrations. Adapted with permission from ref. [78]. Copyright [2020] John Wiley and Sons.

Light from a tapered optical fiber coupled into a microsphere or a microbead can excite optical modes popularly known as whispering gallery modes (WGMs), which are confined at the interface between two media resulting in optical resonance. The sensing mechanism is based on evanescent interaction of the WGM radiation with the surrounding medium leading to a shift in the resonant wavelength (typically observed as a dip in the transmitted light spectrum) [74–76]. The shift in the resonance is typically interrogated using a tunable narrow linewidth laser diode and a photodetector, as illustrated in Figure 6(a).

The minimum angular frequency shift ( ${\left|\Delta \omega \right|}_{\min}$ ) in the resonance angular frequency (*ω*_r_) that can be observed in a reliable manner determines the limit of detection (LoD) in this technique. Such a frequency shift with respect to the cavity resonance frequency is inversely proportional to the resonator quality factor, *Q* and is given by Eq. (5) [79].

(5) $$\frac{{\left|\Delta \omega \right|}_{\min}}{\omega_{\text{r}}}=\frac{F}{Q}$$

where, *F* is the measurement acuity factor. As expected from the physics and illustrated through this simplistic expression, ultra-high *Q* resonators provide ultra-narrow resonances which translates to a low value of frequency shift and hence low LoD values in cases where the small values of frequency shift can be measured with ultrahigh precision.

WGM-based biosensors have proven to be a highly sensitive way of detecting ultra-small virus particles. For example, a WGM biosensor has been demonstrated by Dantham et al. [77] for label-free detection and sizing of the smallest single RNA virus MS2 having a mass of only 6 attogram. This has been achieved using a microsphere exhibiting a *Q* factor (operating wavelength/linewidth) of 4 × 10^5^ placed in a microfluidic cell, and has a nanoplasmonic receptor placed at its equator. MS2 virus particles with a concentration of 330 fM are injected into the microfluidic cell along with 60 mM concentration of salt to aid adsorption. Clear resonance wavelength shifts are observed because of MS2 viruses adsorption with a maximum shift of 17 fm (femto meter) in a 2 fm r.m.s (root mean square) background noise with S/N = 8 (signal-to-noise ratio) (Figure 6(b)). This corresponds to 68 times of enhancement in contrast to the 0.25 fm of wavelength shift due to the adsorption of the virus on a bare micro resonator without nanoplasmonic enhancement.

The virus size is obtained using the expression derived from the FEM model analysis of wavelength enhancement (virus on the hot spot to that on the equator) as a function of virus radius and is found to be 13.3 nm. From FEM analysis, a considerable wavelength shift is observed for decrease in size of the adsorbate, which shows the suitability of the sensor to detect tiny particles. The LoD of the sensor at this r.m.s noise of 2 fm is found to be 5.7 nm viral radius (0.4 attogram mass) and the authors propose that a 2 nm radius bioparticle may be detected by reducing the noise to 0.2 fm with reference interferometer. In summary, by using a single dipole-stimulated plasmonic-nanoshell, detection of the smallest single RNA virus is achieved due to ∼70× microcavity wavelength shift enhancement. This work also suggests that sensitivity can be improved further by using plasmonic nano-rods [77].

In other work, Yue et al. [78] have shown the use of a self-assembled WGM microsphere for sensitive and rapid detection of COVID-19 virus infection through capture of IgG and IgM antibodies. The COVID-19 N-protein decorated silica microsphere shows a linear red-shift with respect to time upon N-protein-IgG binding and the shift rate is proportional to the concentration of the antibody ranging from 1 μg/mL to 100 μg/mL (Figure 6(c)). However, the LoD in their study was only 1000 ng/mL; this low sensitivity is attributable to the relatively low *Q* (∼1000) of their microresonator, presumably due to background scattering [78].

## 4 Comparison of various optical transduction mechanisms

This section compares the performance of various optical diagnostic technologies, including those discussed in the previous section. Table 1 depicts the comparison in terms of Limit of Detection (LoD), sensitivity, specificity and detection time.

**Table 1:** Performance of various optical diagnostic technologies for virus detection.

Method	LoD	Target analyte	Diagnostic Sensitivity	Specificity	Response time	References
Amplitude modulated sensors						
Arrayed imaging reflectometry	125 pM	SARS CoV-2	80%	100%	5 ms	[80, 81]
Colorimetric method	141 nM	SARS CoV-2	96.6%	100%	5 min	[82]
Fluorescence method	35 nM	SARS CoV-2	90%	100%	5 min	[83]
Wavelength modulated sensors						
Fiber Bragg gratings	500 pM	Protein Con A	100%	100%	10 s	[66, 72]
Whispering gallery modes	330 fM	MS-2 virus	100%	100%	20 ms	[25, 77, 78]
Surface plasmon resonance	10 aM	miRNA	94.53%	>97%	<10 s	[32, 84]
Opto-fluidic nano-plasmonic	7 pM	AFP and PSA	100%	100%	1 s	[35, 36, 40]
Surface-enhanced Raman scattering	25 aM	SARS CoV-2	91.4%	83%	10 s	[48, 52]
FTIR/nano-FTIR	35 nM	SARS CoV-2	97%	98.3%	23 s	[60, 63, 85]

The assay (diagnostic) sensitivity in Table 1 is expressed in percentage based on the true positive rate of the number of samples used for viral detection. Compared with amplitude/intensity modulated sensors, the wavelength modulated sensors perform better in terms of sensitivity. WGM [77, 86], FBG [66, 72] and nano-plasmonic [35, 36, 40] methods are reported to have excellent assay sensitivity. A review analysis of WGM techniques reports that ultimate specificity and sensitivity can be obtained through a proper choice of bio-recognition element. The functionalized layer on the fiber in FBG method traps the virus particles producing a change in RI of the surrounding medium improving the assay sensitivity. A novel combination of plasmonic sensing and nanofluidics has led to higher sensitivity. Hybrid WGM-plasmonic techniques [87–90] have been reported with excellent wavelength sensitivity. FBG coupled with plasmonic, long period grating (LPG) and LPFG sensors [91–93] too provide excellent wavelength sensitivity, but not explored in biosensing. LoD is expressed in molar concentration of the analytes used in these techniques. The best LoD values reported in the literature for various virus/bio molecules species are listed in Table 1. We anticipate that the LoD values can be extended to SARS CoV-2 as well. However, please note that the values do not represent the fundamental limits of the different techniques as they strongly depend on various factors including the surface functionalization, the choice of nano-materials coated, background losses, quality factor of any resonator structure, the surrounding environment, and measurement limit of the optical interrogator e.g. optical spectrum analyzer (OSA) used for logging data. Multiple references are used to provide complete data in Table 1. Although the LoD values are reported in different units in literature, we have converted them into molar units for straightforward comparison. This was done by using the NIBSC conversion factor [94, 95] of 1 IU/mL = 1.72 copies/mL and dividing the value of IU by 0.83 to convert into ng/mL, before finally converting them into molar values. 

There are several other reports on the detection of virus with high sensitivity and specificity. Kitane et al. [60] developed a machine learning classification of ATRFTIR and reported a high specificity of 97% from 280 samples (100 positive and 180 negative). Nano-FTIR based infrared spectra of samples exhibit as low as 20 nm spatial resolution [54]. Structural analysis of protein complexes [63] demonstrates single protein sensitivity with nanoscale resolution. The localized SPR [84] method utilizes the photothermal effect at plasmonic resonance frequency for discriminating specific gene sequence in a multi-gene mixture. A derivative SERS spectrum along with principal component analysis with linear discriminant analysis (PCA-LDA) diagnostic algorithm has clearly differentiated the virus from healthy samples [48].

Specificity measures the true negative rate among the number of samples used for viral detection. The proper choice of antibody in WGM shows high affinity and specificity towards the antigen. The GO surface with oxygenated functional groups in FBG method provides an effective binding site for the probe surface resulting in 100% specificity. The intensity and wavelength changes are observed for different virus densities. High specificity was achieved in LSPR method through extensive nonspecific binding tests. A statistical analysis by Lu et al. [48] of SERS spectrum differentiates healthy volunteers from hepatitis-B patients with a specificity of 83%. They report a specificity of 91.4% with 187 samples (93 positive and 94 negative) using SERS method.

The time taken to detect the presence of a virus after it has bound to the surface of the sensing platform is another key parameter. Please note that the detection time mentioned in Table 1 are readout times instead of sample-to-result time. The detection time of WGM is one of the least to detect the presence of virus. Tracing a single molecule in sample needs few seconds in WGM method, although the virus detection itself is only in the order of milliseconds due to the ultrasmall cavity structure. Spectra were acquired in 1000 ms in opto-fluidic nano-plasmonic method [36]. A rapid increase [96] in fluorescence intensity in an LSPCF biosensor leads to a relatively fast SARS coronavirus detection. Increasing the exposure time in FBG method increases the sensitivity of the detection, which in turn determines the level of illness. Nano-FTIR records the interferograms [85] in 23 s.

Considering the parameters reported in Table 1, the transduction mechanisms that are well suited for fast virus detection are: WGM, SPR, SERS, FBG, and nano-FTIR.

## 5 Future prospects of nanophotonic biosensors for virus detection

Although excellent progress has been made towards highly sensitive and robust virological species detection by optical diagnostic techniques (as summarized above), there is tremendous scope for further developments in this field leading to cost-effective, lab-on-a-chip solutions that exhibit ultrahigh sensitivity with rapid detection speed and mass-manufacturability.

In general, point-of-care diagnostics developed within an Internet of Things (IoT) framework for seamless and secure data transfer using smartphones to enable real-time decision making is of much importance in the present-day scenario. With rapid developments in microfluidics, micro-electro-mechanical systems (MEMS), nanotechnology and material science, there is tremendous scope for low cost point-of-care diagnostics, which are likely to be dominated by optical technologies in the near future [97]. In the discussion below, we analyze a variety of optical sensors in terms of their anticipated potential by focusing on key parameters of interest (such as sensitivity, speed, reliability, standoff/remote detectability, and manufacturability).

**Projected Improvements to Measurement Sensitivities and LoDs of Key Photonic Biosensors:** As discussed earlier, the optical resonant field enhancement through methods such as whispering gallery modes (WGM), nano-FTIR, and plasmons or polaritons pose exciting pathways to enhance the viral measurement sensitivities. Specifically, by attaching a plasmonic metal nano-rod to a WGM sensor, detection of a single protein molecule (10 nm size) has been recently demonstrated [98], albeit by a method that is relatively slow and somewhat difficult to implement routinely. Nevertheless, such work has opened up interesting possibilities, including the study of structural dynamics in individual protein molecules. Although localized plasmons are much preferred due to the strong enhancement of the local electric field, their sensitivity is an order of magnitude lower than their guided counterparts [99]. In order to improve their sensitivity, a plasmonic metamaterial capable of supporting a guided mode in a porous nanorod layer may be used. Recent reviews in this topic [100] have indicated their potential to be game changers in the field of label-free virus detection. A major future goal is to explore the possibility of using various optical resonant enhancement techniques to determine the applicability of each technique for specific viruses of interest, and to estimate the measurement sensitivity of each technique, in terms of picomolar or attomolar concentrations that may be measurable for target molecules of interest.

It is interesting to also note that there have also been attempts to demonstrate quantum sensing [101] through strong coupling between WGMs and single nitrogen-vacancy (NV) centers in diamond nanocrystals. Although such a quantum sensing approach seems feasible, there are several practical challenges to be overcome before they can be implemented in commercial applications. One of the key challenges is to isolate such sensitive quantum events from thermo-acoustic noise due to external environment. To this end, the statistics of the detection has to be quantified using tools such as receiver operating characteristic (ROC) curves and area under the curve (AUC) [102]. Finally, such quantum sensors have to be realized at a reasonable price and at practical operating temperatures to become commercially viable; nevertheless, this methodology may become applicable to practical viral sensing experiments in the foreseeable future. In recent years, graphene and related 2D materials have been explored for biosensing and healthcare applications. 2D materials have exceptional optical biosensing properties such as excellent biocompatibility, large surface-to-volume ratios, ultrafast carrier mobilities, exceptional fluorescence-quenching abilities, broadband light absorption, high chemical stability, and outstanding robustness and flexibility. The most striking features of optical biosensors based on 2D materials are ultrahigh sensitivity and ultrafast response times [103].

**Projected Improvements to Sensing Speeds/Faster Response Times of Key Photonic Biosensors:** Another desirable characteristic for an ideal sensor is the rapid and robust detection of viral particles. From the perspective of speed of detection, FBG-based sensors are attractive since their response time is not limited by cavity lifetime issues associated with resonator configurations (e.g., WGM sensors). However, because of the ultrasmall cavity sizes, microresonator-based WGM sensors have already achieved response times of the order of nanoseconds, which is generally sufficient to study structural dynamics of single protein molecules [104] in a label-free environment.

**Projected Improvements in Mass Manufacturability of Key Photonic Biosensors:** In spite of rapid advances in the performance metrics of optical diagnostics for virus detection, widespread penetration of optical diagnostic methods in the marketplace would primarily depend on the economies of scale. This relates to the amenability of mass manufacturing of the biosensors through a simple and reliable processes. One interesting possibility is the development of a lab-on-a-chip (LOC) on silicon platform with appropriate microfluidic channels for transportation, mixing, detection, and collection [105] of various components of the sample during the biodetection process. Miniaturization of such immunoassays can potentially lead to reduced test times, reduced sample volumes, parallel processing (increased data collection), and increased portability. In fact, this may also facilitate simultaneous multivirus detection or genome sequencing with massively parallel immunoassays (1000s of individual cells). Microwells can be etched in Si substrates, which may subsequently be functionalized with different biomolecules to detect multiple analytes simultaneously; the related electronics for the detection may also be integrated onto the same substrate, thereby realizing highly desirable “lab-on-a-chip” type of capabilities.

In general, the above-described biosensors may be used to analyze a wide range of analytes including toxicants, drugs, pesticides, biomarkers, heavy metals, pathogens, disease markers etc. in different sample formats (extracts, body fluids, tissue, water, soil, food, etc.) for many applications such as environmental monitoring, disease detection, food safety, defence, and drug discovery. Beyond the nanophotonic technologies described here, one of the key enablers for these applications is the appropriate surface functionalization of the appropriate biosensors platforms, as discussed above.

## 6 Summary

Viruses are a significant source of diseases that are highly contagious. Viral diseases can be transmitted through bodily fluids, water, and air. In order to control the spread of viral diseases – especially during pandemics – accurate, affordable, and rapid nanophotonics diagnostic methods are critically needed to test and monitor large numbers of people. Currently, significant efforts are being invested towards developing point-of-care devices that enable rapid and accurate diagnosis of viruses and viral infections. One such test based on polymerase chain reaction (PCR) provides the required specificity and sensitivity for COVID-19 detection; however, it takes a relatively long time to provide the final result, which delays large scale detection and control. The alternative point-of-care diagnostic technique is a rapid lateral flow test (serological and antigen tests), which can provide the result in a few minutes, but often falls short of the required sensitivity. Such limitations could be overcome by label-free, highly sensitive, and rapid methods for the detection of highly contagious virus species (such as COVID-19). Optical biosensing or nano-biophotonic techniques seem to be very promising in this regard, and the development of point-of-care diagnostics instruments based on commercially viable optical biosensing techniques are expected in the foreseeable future.
